# Clinical characteristics and risk factors associated with ICU-acquired infections in sepsis: A retrospective cohort study

**DOI:** 10.3389/fcimb.2022.962470

**Published:** 2022-07-28

**Authors:** Yajun He, Jiqian Xu, Xiaopu Shang, Xiangzhi Fang, Chenggang Gao, Deyi Sun, Lu Yao, Ting Zhou, Shangwen Pan, Xiaojing Zou, Huaqing Shu, Xiaobo Yang, You Shang

**Affiliations:** ^1^ Department of Critical Care Medicine, Union Hospital, Tongji Medical College, Huazhong University of Science and Technology, Wuhan, China; ^2^ Institute of Anesthesiology and Critical Care Medicine, Union Hospital, Tongji Medical College, Huazhong University of Science and Technology, Wuhan, China; ^3^ Department of Information Management, Beijing Jiaotong University, Beijing, China

**Keywords:** sepsis, ICU-acquired infection, risk factors, prediction, MIMIC database

## Abstract

Intensive care unit (ICU)-acquired infection is a common cause of poor prognosis of sepsis in the ICU. However, sepsis-associated ICU-acquired infections have not been fully characterized. The study aims to assess the risk factors and develop a model that predicts the risk of ICU-acquired infections in patients with sepsis.

**Methods:**

We retrieved data from the Medical Information Mart for Intensive Care (MIMIC) IV database. Patients were randomly divided into training and validation cohorts at a 7:3 ratio. A multivariable logistic regression model was used to identify independent risk factors that could predict ICU-acquired infection. We also assessed its discrimination and calibration abilities and compared them with classical score systems.

**Results:**

Of 16,808 included septic patients, 2,871 (17.1%) developed ICU-acquired infection. These patients with ICU-acquired infection had a 17.7% ICU mortality and 31.8% in-hospital mortality and showed a continued rise in mortality from 28 to 100 days after ICU admission. The classical Systemic Inflammatory Response Syndrome Score (SIRS), Sequential Organ Failure Assessment (SOFA), Oxford Acute Severity of Illness Score (OASIS), Simplified Acute Physiology Score II (SAPS II), Logistic Organ Dysfunction Score (LODS), Charlson Comorbidity Index (CCI), and Acute Physiology Score III (APS III) scores were associated with ICU-acquired infection, and cerebrovascular insufficiency, Gram-negative bacteria, surgical ICU, tracheostomy, central venous catheter, urinary catheter, mechanical ventilation, red blood cell (RBC) transfusion, LODS score and anticoagulant therapy were independent predictors of developing ICU-acquired infection in septic patients. The nomogram on the basis of these independent predictors showed good calibration and discrimination in both the derivation (AUROC = 0.737; 95% CI, 0.725–0.749) and validation (AUROC = 0.751; 95% CI, 0.734–0.769) populations and was superior to that of SIRS, SOFA, OASIS, SAPS II, LODS, CCI, and APS III models.

**Conclusions:**

ICU-acquired infections increase the likelihood of septic mortality. The individualized prognostic model on the basis of the nomogram could accurately predict ICU-acquired infection and optimize management or tailored therapy.

## Background

Sepsis is characterized by an injurious host response against various severe infections and is among the most serious medical conditions worldwide, leading to high mortality rates of patients in the intensive care unit (ICU) (Rudd et al., 2020; Cavaillon et al., 2020). Among the deaths from sepsis, approximately 15% are acute, occurring soon after sepsis onset, while 85% are late phase, usually due to ICU-acquired infections (Nedeva et al., 2020). Currently, the dominant view on the underlying causes of these deaths is that the patients with sepsis experience immunosuppression (Stortz et al., 2018). Immunosuppressed patients are highly susceptible to dangerous ICU-acquired opportunistic pathogens and can rapidly succumb to infection (Sundar and Sires, 2013; Koch et al., 2017). The ICU-acquired infections are significantly associated with increased in-hospital mortality, length of stay (LOS), and poor prognosis (Otto et al., 2011; van Vught et al., 2017). Therefore, it is necessary to identify the risk factors of ICU-acquired infections in patients with sepsis.

In patients with sepsis, this risk of developing ICU-acquired infection is influenced by multiple interrelated factors. For example, some studies from 10 years ago with small sample sizes or covering a short period of (Chen et al., 2022) time showed that advanced age, disease severity, deep venous catheterization, and invasive ventilation were associated with an increased risk of incident ICU-acquired infection in sepsis (Wolkewitz et al., 2014; van Vught et al., 2016; Chen et al., 2019). Despite associations of lower human leukocyte antigen expression and increased interleukin-10 (IL-10) levels with ICU-acquired infection (van Vught et al., 2017; Stortz et al., 2018), identifying which patients are at a higher risk of sepsis-associated ICU-acquired infection remains clinically challenging. Currentlly, many decisions surrounding the management of sepsis-associated ICU-acquired infection mainly rely on accurate estimates of a patient’s future likelihood of developing infection (van Vught et al., 2016). However, sepsis-associated ICU-acquired infections have yet to be fully characterized.

Here, we analyzed the data from the large public database Medical Information Mart for Intensive Care (MIMIC) IV and attempted to identify the differences in clinical characteristics between septic patients who did and did not experience ICU-acquired infection, identify the risk factors associated with septic ICU-acquired infection occurrence and its outcomes, and develop a novel risk prediction model that predicts ICU-acquired infections for patients with sepsis.

## Methods

### Database

All of the clinical data analyzed in this study were extracted from the online international critical care database MIMIC-IV (version 1.0) (Johnson et al., 2018), which comprises patient-related data collected from the critical care units at the Beth Israel Deaconess Medical Center between 2008 and 2019. This is a large longitudinal database, including data from 382,278 patients and 523,740 admissions in critical care. One of the authors of this study (YH) has completed the Collaborative Institutional Training Initiative examination (Certification number: 31935546). The code used for data extraction is available on GitHub (https://github.com/MIT-LCP/mimic-iv).

### Study population and definitions

The definition of sepsis was referred to in the Third International Consensus Definitions for Sepsis and Septic Shock (Sepsis-3), which means that patients had a suspected infection and a Sequential Organ Failure Assessment (SOFA) score ≥2 (Singer et al., 2016). Patients younger than 18 years, with SOFA score <2 at ICU admission, and with ICU stay less than 48 h were excluded (van Vught et al., 2016). Initial ICU-acquired infection was defined as any new-onset possible infection with new positive pathogenic microorganism culture and a new antibiotic regimen after 48 h from ICU admission according to the Centers for Disease Control and Prevention criteria (Singer et al., 2016; Johnson et al., 2018).

### Data collection

Only patients with sepsis on the first ICU admission who met the inclusion criteria were included in the analysis to avoid double counting of repeat admission. Patient baseline parameters were extracted from the database as follows: age, sex, weight, LOS in ICU and hospital, and death. Basic ICU rating scales at admission, such as SOFA, Systemic Inflammatory Response Syndrome Score (SIRS), Oxford Acute Severity of Illness Score (OASIS), Logistic Organ Dysfunction Score (LODS), Simplified Acute Physiology Score II (SAPS II), Acute Physiology Score III (APS III), and Charlson Comorbidity Index (CCI), were calculated and collected at admission. Comorbidities on the first admission included shock, cardiovascular and cerebrovascular disease, surgery, cancer, renal disease, and liver insufficiency. Intervention events included pharmacy (vasopressors, glucocorticoids, heparin), transfusion of blood, mechanical ventilation, renal replacement therapy, central venous catheter, and urinary catheter. Laboratory information was collected at ICU admission. Microbial test results recorded in the database were used as evidence of ICU-acquired infection. The time of ICU-acquired infection occurrence and all pathogenic microorganisms and infected sites from initiation to ICU discharge were extracted.

### Statistical analysis

Variables with >20% missing values were excluded, and the remaining missing data were estimated using multiple imputations. For descriptive data, parametric quantitative variables are expressed as mean and standard deviation, nonparametric quantitative variables as the median and interquartile range (IQR), and categorical variables as number and percentage. Differences between patients with and without ICU-acquired infection were analyzed using the *t*-test or chi-square test for parametric variables, Mann–Whitney U or Kruskal–Wallis test for continuous nonparametric variables, and Fisher’s exact test for categorical variables. Kaplan–Meier survival curve and landmark analyses using the log-rank test were applied to estimate the survival rates. All patients were randomly assigned to the training or validation cohorts based on a ratio of 7:3 (Yang et al., 2021; Liu et al., 2021). In the training cohort, potential prognostic factors were screened out using univariate regression that may be related to ICU-acquired infection, including all variables with a p-value <0.05. Then, the factors were further analyzed using a binary multivariate logistic regression model to identify risk factors (Zhang et al., 2018). Subsequently, factors with prognostic significance in the multivariate logistic regression analysis were utilized to build a prediction model, and a nomogram was used to visualize the model. Odds ratio (OR) values were calculated with corresponding 95% confidence intervals (CIs). The discrimination performance of the nomogram was measured using Harrell’s concordance index (C-index), a receiver operating characteristic (ROC) curve, area under the ROC (AUROC) curve, and calibration plots, while the calibration plot was used to graphically evaluate the calibration of the nomogram in both training and validation cohorts. Lastly, the decision curve analysis (DCA) was used to access the clinical usefulness of the new predictive models and scores model for ICU-acquired infection by quantifying the net benefit against threshold probabilities.

Stata/IC 16.0 software (Stata Corp., College Station, TX, USA) was used to conduct statistical analyses. Two-sided p < 0.05 was considered statistically significant. The “TSHRC” and “survminer” packages were used for landmark analysis. The “rms” and “regplot” packages were used to conduct a logistic regression analysis model and for nomogram development. Calibration curves, ROC curves, and DCA curves were generated using the “graphics,” “pROC,” and “rmda” packages in the R programming language (version 4.1.3; R Foundation for Statistical Computing, Vienna, Austria).

## Results

### Demographic and clinical characteristics of sepsis included in this study

**Figure 1:**
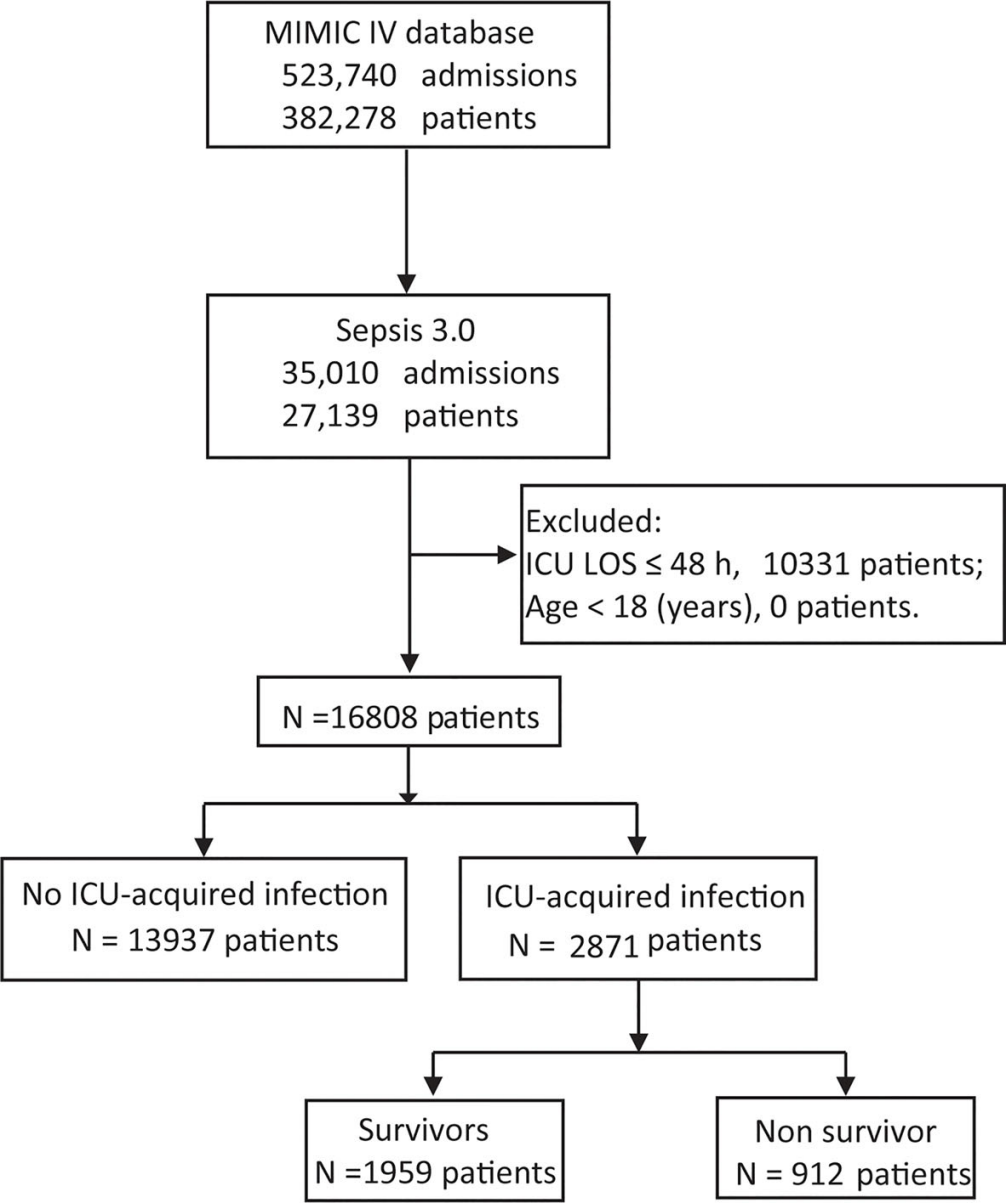
Study design flowchart.

We screened 16,808 patients who fulfilled the inclusion criteria (**Figure 1**). The baseline characteristics of patients on admission are presented in **Table 1**. The mean age of the included patients was 64.65 ± 16.27 years, and 9,609 patients (57.2%) were men. Among patients with sepsis, 2,871 (17.1%) patients were complicated by the ICU-acquired infections. The mean ages were 62.49 ± 16.62 years in the patients with ICU-acquired infections and 65.09 ± 16.17 years in the patients without ICU-acquired infections (p < 0.001). One thousand seven hundred three (59.3%) septic patients with ICU-acquired infections were from the surgical ICU, and the number of the patients without ICU-acquired infections is 6,521 (46.8%).

**Table 1:** Baseline characteristics and outcomes of sepsis stratified according to the development or not of ICU-acquired infection.

Patients	All patients	No ICU-acquired infection	ICU-acquired infection	p-value
	(N = 16,808) (100%)	(N = 13,937) (82.9%)	(N = 2,871) (17.1%)	
**Demographics**				
Age, years (mean, SD)	64.65 (16.27)	65.09 (16.17)	62.49 (16.62)	<0.001
Age <60 years (%)	5,734 (34.1%)	4,629 (33.2%)	1,105 (38.5%)	<0.001
Gender, men (%)	9,609 (57.2%)	7,975 (57.2%)	1,634 (56.9%)	0.761
BMI, kg/m^2^ (mean, SD)	29.09 (7.92)	28.99 (7.82)	29.49 (8.30)	0.008
BMI, >25 kg/m^2^	7,512 (68.1%)	5,948 (67.8%)	1,564 (69.1%)	0.238
**Admission**				
** Admission ICU unit (%)**				
Medical	8,584 (51.1%)	7,416 (53.2%)	1,168 (40.7%)	<0.001
Surgical	8,224 (48.9%)	6,521 (46.8%)	1,703 (59.3%)	<0.001
** Severity of disease**				
SOFA (mean, SD)	3.76 (2.13)	3.70 (2.07)	4.05 (2.39)	<0.001
SIRS (mean, SD)	2.83 (0.89)	2.81 (0.89)	2.90 (0.91)	<0.001
LODS (mean, SD)	6.40 (3.40)	6.07 (3.28)	8.00 (3.51)	<0.001
SAPS II (mean, SD)	40.92 (13.90)	40.63 (13.63)	42.31 (15.04)	<0.001
APS III (mean, SD)	59.28 (25.58)	56.83 (24.51)	71.17 (27.28)	<0.001
OASIS (mean, SD)	36.47 (9.02)	35.74 (8.88)	40.02 (8.89)	<0.001
CCI (mean, SD)	6.01 (2.97)	6.05 (2.96)	5.82 (2.99)	<0.001
** Chronic comorbidity (%)**				
Cerebrovascular insufficiency	884 (5.3%)	632 (4.5%)	252 (8.8%)	<0.001
Cardiovascular insufficiency	3,811 (22.7%)	3,320 (23.8%)	491 (17.1%)	<0.001
Respiratory insufficiency	480 (2.9%)	413 (3.0%)	67 (2.3%)	0.065
Liver insufficiency	1,472 (8.8%)	1,214 (8.7%)	258 (9.0%)	0.634
Renal insufficiency	403 (2.4%)	337 (2.4%)	66 (2.3%)	0.704
Cardiovascular shock	1,144 (6.8%)	888 (6.4%)	256 (8.9%)	<0.001
Sepsis shock	3,887 (23.1%)	3,064 (22.0%)	823 (28.7%)	<0.001
Trauma shock	117 (0.7%)	70 (0.5%)	47 (1.6%)	<0.001
** Treatment interventions before ICU-acquired infections events (%)**				
Any hydrocortisone use	15,293 (91.0%)	12,834 (92.1%)	2,459 (85.6%)	<0.001
Hydrocortisone >200 mg (2d)^a^	2,001 (11.9%)	1,573 (11.3%)	428 (14.9%)	<0.001
Norepinephrine, median (IQR), mg	0.0 (0.0, 2,576.7)	0.0 (0.0, 1,891.7)	0.0 (0.0, 7,276.3)	<0.001
Anticoagulant	10,311 (61.3%)	8,167 (58.6%)	2,144 (74.7%)	<0.001
Any norepinephrine use (2d)^b^	15,873 (94.4%)	13,427 (96.3%)	2,446 (85.2%)	<0.001
RBC	6,641 (39.5%)	5,157 (37.0%)	1,484 (51.7%)	<0.001
Cell Saver Intake	1,950 (11.6%)	1,670 (13.0%)	280 (7.1%)	<0.001
Cryoprecipitate	639 (3.8%)	450 (3.2%)	189 (6.6%)	<0.001
Central venous catheter	9,684 (57.6%)	7,682 (55.1%)	2,002 (69.7%)	<0.001
Mechanical ventilation	9,851 (58.6%)	7,697 (55.2%)	2,154 (75.0%)	<0.001
Urinary catheter	4,421 (26.3%)	3,240 (25.2%)	1,181 (29.7%)	<0.001
Tracheostomy	76 (0.5%)	46 (0.3%)	30 (1.0%)	<0.001
Renal replacement therapy	612 (3.6%)	431 (3.1%)	181 (6.3%)	<0.001
Primary infection of sepsis (%)^c^				
Primary infection	6,151 (36.6%)	4,924 (35.3%)	1,227 (42.7%)	<0.001
Causative Pathogen, No. (%)				
Gram-positive bacteria	3,573 (21.3%)	2,887 (20.7%)	686 (23.9%)	<0.001
* Staphylococcus aureus*	712 (4.2%)	572 (4.1%)	140 (4.9%)	0.061
MRSA	498 (3.0%)	395 (2.8%)	103 (3.6%)	0.030
* Streptococcus pneumoniae*	521 (3.1%)	418 (3.0%)	103 (3.6%)	0.098
Gram-negative bacteria	2,360 (14.0%)	1,839 (13.2%)	521 (18.1%)	<0.001
* Escherichia coli*	330 (2.0%)	274 (2.0%)	56 (2.0%)	0.957
* Pseudomonas* species	102 (0.6%)	67 (0.5%)	35 (1.2%)	<0.001
* Klebsiella* species	128 (0.8%)	98 (0.7%)	30 (1.0%)	0.055
VRE	151 (0.9%)	125 (0.9%)	26 (0.9%)	0.964
* Notrophomonas maltophilia*	23 (0.1%)	15 (0.1%)	8 (0.3%)	0.024
* Acinetobacter baumannii*	11 (0.1%)	6 (0.0%)	5 (0.2%)	0.012
Virus	113 (0.7%)	87 (0.6%)	26 (0.9%)	0.093
Fungi	1,731 (10.3%)	1,357 (9.7%)	374 (13.0%)	<0.001
* Candida*	55 (0.3%)	47 (0.3%)	8 (0.3%)	0.617
* Aspergillus*	25 (0.1%)	21 (0.2%)	4 (0.1%)	0.886
Source of Infection, No. (%)				
Skin	687 (4.1%)	546 (3.9%)	141 (4.9%)	0.014
Tissue	923 (5.8%)	743 (5.6%)	180 (6.9%)	0.009
Pulmonary tract	2,852 (17.0%)	2,193 (15.7%)	659 (23.0%)	<0.001
Bloodstream infection	3,441 (20.5%)	2,833 (20.3%)	608 (21.2%)	0.304
Abdomen	137 (0.8%)	110 (0.8%)	27 (0.9%)	0.412
Neurological	108 (0.6%)	91 (0.7%)	17 (0.6%)	0.710
Urinary tract	4,012 (23.9%)	3,400 (24.4%)	612 (21.3%)	<0.001
Stool	340 (2.0%)	291 (2.1%)	49 (1.7%)	0.186
Pleura	88 (0.5%)	72 (0.5%)	16 (0.6%)	0.783
Bile	75 (0.4%)	61 (0.4%)	14 (0.5%)	0.715
Other^c^	11 (0.1%)	6 (0.0%)	5 (0.2%)	0.012
**Outcome**				
**Length of stay, median (IQR), d**				
Hospital	9.9 (6.3, 16.7)	8.9 (6.0, 14.5)	17.6 (11.2, 26.2)	<0.001
ICU	4.8 (3.0, 8.9)	4.1 (2.8, 6.9)	12.1 (7.6, 18.5)	<0.001
** Mortality (%)**				
Hospital	3,512 (20.9%)	2,600 (18.7%)	912 (31.8%)	<0.001
ICU	1,543 (9.2%)	1,036 (7.4%)	507 (17.7%)	<0.001
** Discharge location**				<0.001
Clinical ward	8,681 (51.6%)	7,024 (50.4%)	1,657 (57.7%)	<0.001
Home	5,162 (30.7%)	4,792 (34.4%)	370 (12.9%)	<0.001
Other/Unknown	138 (0.8%)	123 (0.9%)	15 (0.5%)	0.052
Died	2,796 (16.6%)	1,970 (14.1%)	826 (28.8%)	<0.001
** Laboratory parameter**				
WBC, 10⁹/L, median (IQR)	11.8 (8.3, 16.4)	11.7 (8.2, 16.3)	12.0 (8.5, 16.8)	0.106
Lymphocytes, 10⁹/L, median (IQR)	45.5 (1.3, 123.0)	45.1 (1.3, 123.4)	47.3 (1.3, 118.3)	0.616
Lymphocytes <0.7 × 10⁹/L	1,736 (10.3%)	1,436 (10.3%)	300 (10.4%)	0.815
Neutrophils, 10⁹/L, median (IQR)	389.8 (10.6, 1,013.0)	440.8 (11.4, 1,101.7)	495.1 (12.4, 1,165.1)	0.129
Neutropenia (<0.5 × 10⁹/L)	79 (0.5%)	68 (0.5%)	11 (0.4%)	0.455
Monocytes, 10⁹/L	19.2 (0.9, 54.0)	18.6 (0.9, 53.8)	23.1 (0.9, 54.3)	0.792
Platelets, 10⁹/L, median (IQR)	187.0 (130.0, 258.0)	185.0 (129.0, 256.0)	197.0 (131.0, 267.5)	<0.001
Platelets <50 × 10⁹/L	889 (5.3%)	719 (5.2%)	170 (5.9%)	0.097
AST, U/L, median (IQR)	40.0 (24.0, 86.0)	40.0 (24.0, 83.0)	37.0 (24.0, 78.5)	0.824
ALT, U/L, median (IQR)	27.0 (16.0, 57.0)	27.0 (16.0, 52.0)	26.0 (15.0, 54.0)	0.344
ALT, <40 U/L	6,773 (40.3%)	5,519 (39.6%)	1,254 (43.7%)	<0.001
AST, <40 U/L	5,207 (31.0%)	4,301 (30.9%)	906 (31.6%)	0.462
PaO_2_/FiO_2_, mmHg, median (IQR)	302.0 (204.0, 403.3)	299.0 (202.0, 400.0)	310.0 (210.0, 420.0)	<0.001
PaO_2_/FiO_2_ <300 mmHg	12,169 (72.4%)	10,235 (73.4%)	1,934 (67.4%)	<0.001
PaO_2_/FiO_2_ <200 mmHg	3,243 (19.3%)	2,592 (18.6%)	651 (22.7%)	<0.001
INR	1.3 (1.2, 1.6)	1.3 (1.2, 1.6)	1.3 (1.1, 1.6)	0.578
PT, s, median (IQR)	14.6 (12.8, 17.5)	14.6 (12.8, 17.5)	14.3 (12.6, 17.6)	0.316
APTT, s, median (IQR)	31.3 (27.4, 38.8)	31.4 (27.4, 38.5)	31.3 (27.2, 41.1)	0.05
BUN, mg/dl, median (IQR)	21.0 (14.0, 35.0)	21.0 (14.0, 35.0)	21.0 (14.0, 35.0)	0.719
Calcium, mg/dl, median (IQR)	8.2 (7.7, 8.8)	8.2 (7.7, 8.7)	8.2 (7.7, 8.8)	0.982
Creatinine, mg/dl, median (IQR)	1.0 (0.7, 1.6)	1.0 (0.7, 1.6)	1.0 (0.7, 1.6)	0.232

ICU, intensive care unit; SD, standard deviation; IQR, interquartile range; BMI, body mass index; LODS, Logistic Organ Dysfunction System; APS III, Acute Physiology Score III; OASIS, Oxford Acute Severity of Illness; SIRS, Systemic Inflammatory Response Syndrome Score; SAPS II, Simplified Acute Physiology Score II; SAPS II, Simplified Acute Physiology Score II; SOFA, Sequential Organ Failure Assessment; CCI, Charlson Comorbidity Index; RBC, red blood cell; VRE, vancomycin-resistant enterococci; MRSA, methicillin-resistant Staphylococcus aureus; LOS, length of stay; WBC, white blood cell; SpO_2_, peripheral blood oxygen saturation; ALT, alanine aminotransferase; AST, aspartate transaminase; BUN, blood urea nitrogen; PT, partial thromboplastin time; APTT, activated partial thromboplastin time.

a The use of any hydrocortisone or its equivalent (hydrocortisone dose = 4× prednisolone dose, 5× methylprednisolone dose, 25× dexamethasone dose).

b The use of noradrenaline for >24 h at a dose of >0.1 µg/kg/min in the first 48 h in the ICU.

c The site of infection and pathogenic microorganisms of all sepsis patients recorded within 48 h of ICU admission.

d Other infection indicates unspecified sites other than those listed.

Moreover, there was a higher prevalence of cardiovascular shock [256 (8.9%) vs. 888 (6.4%), p < 0.001], septic shock [823 (28.7%) vs. 3,064 (22.0%), p < 0.001], trauma shock [47 (1.6%) vs. 117 (0.7%), p < 0.001], and cerebrovascular insufficiency [252 (8.8%) vs. 632 (4.5%), p < 0.001] in the patients with ICU-acquired infections.

The primary infection of sepsis included Gram-positive bacteria (21.3%) and Gram-negative bacteria (14.0%). In comparison with patients without ICU-acquired infections, patients with ICU-acquired infections have a higher rate of Gram-positive bacteria infections [686 (23.9%) vs. 2,887 (20.7%), p < 0.001], especially *methicillin-resistant Staphylococcus aureus* (MRSA) infections [103 (3.6%) vs. 395 (2.8%), p = 0.03], and Gram-negative bacteria [521 (18.1%) vs. 1,839 (13.2%), p < 0.001] infections, such as *Pseudomonas* species infection [35 (1.2%) vs. 67 (0.5%), p < 0.001], *Acinetobacter baumannii* [5 (0.2%) vs. 6 (0.0%), p = 0.012], and *Notrophomonas maltophilia* [8 (0.3%) vs. 15 (0.1%), p = 0.024]. The patients with pulmonary tract infection [659 (23.0%) vs. 2,193 (15.7%), p < 0.001] on admission were more likely to develop ICU-acquired infections. There were no significant differences in leukocytes, lymphocytes, neutrophils, and coagulation function on ICU admission between the two groups (**Table 1**).

### Characteristics of intensive care unit-acquired infections

The ICU-acquired infection sites and pathogens are presented in **Table 2**. The median time from admission to the first ICU-acquired infection was 4.1 days (IQR, 2.9–6.5). The common sites in the patients with ICU-acquired infection included respiratory tract (n = 1,442, 50.2%), urinary tract (n = 717, 25%), and catheter-related bloodstream (n = 385, 13.4%). The common causative pathogens were Gram-negative bacteria (1,152, 40.2%), Gram-positive bacteria (1,154, 40.1%), and fungi (n = 544, 18.9%) in the patients with ICU-acquired infection.

**Table 2:** Characteristics of ICU-Acquired Infections After Admission for Sepsis.

**ICU-Acquired Infections**	(N = 2871)
Day of first ICU-acquired infection,days,median (IQR)	4.1 (2.9, 6.5)
**Source of infection**	
Pulmonary	1,442 (50.2%)
Urinary tract	717 (25.0%)
Catheter-related bloodstream infection	385 (13.4%)
Abdominal infection	37 (1.3%)
Skin	96 (3.3%)
Stool	153 (5.3%)
Pleura	13 (0.5%)
Bile	14 (0.5%)
Tissue	139 (4.8%)
**Causative Pathogen, No. (%)**	
Gram-negative bacteria	1,154 (40.2%)
Gram-positive bacteria	1,152 (40.1%)
Fungi	544 (18.9%)
Viruses	22 (0.8%)

### Outcomes of septic patients with intensive care unit-acquired infections

The patients with ICU-acquired infections had longer hospital LOS [17.6, IQR 11.2–26.2 vs. 8.9, IQR 6.0–14.5; p < 0.001) and ICU LOS (12.1, IQR 7.6–18.5 vs. 4.1, IQR 2.8–6.9; p < 0.001) than those without it. Moreover, the septic patients with acquired infection had higher ICU mortality [507 (17.7%) vs. 1,036 (7.4%); p < 0.001] and hospital mortality [912 (31.8%) vs. 2,600 (18.7%); p < 0.001] than those without it (**Table 1**). One thousand six hundred fifty-seven (57.7%) septic patients with ICU-acquired infections were assigned to the clinical ward after discharge; only 370 (12.9%) patients were able to return home after discharge.

**Figure 2:**
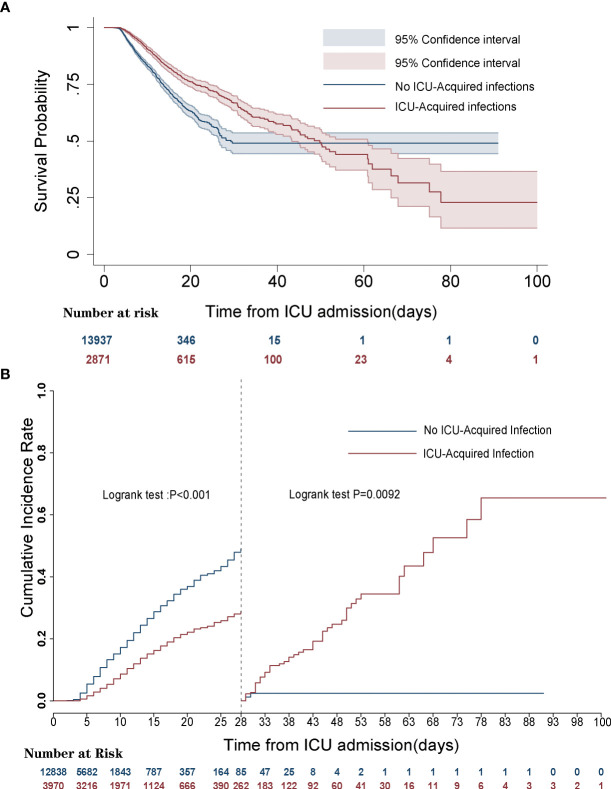
Survival curves of septic patients with and without intensive care unit (ICU)-acquired infections. **(A)** Kaplan–Meier survival curve. **(B)** Landmark analyses discriminating cumulative mortality comparing ICU-acquired infections with no ICU-acquired infections.

Kaplan–Meier survival curve and landmark analysis depicted the significantly higher 28-day mortality for the patients without ICU-acquired infections compared with that of patients with acquired infection, but there was a consistently higher mortality from 28 days to 100 days after ICU admission in ICU-acquired infections (log-rank p < 0.001) (**Figure 2**).

### Prognostic factors associated with intensive care unit-acquired infection in sepsis and construction of the nomogram

**Figure 3:**
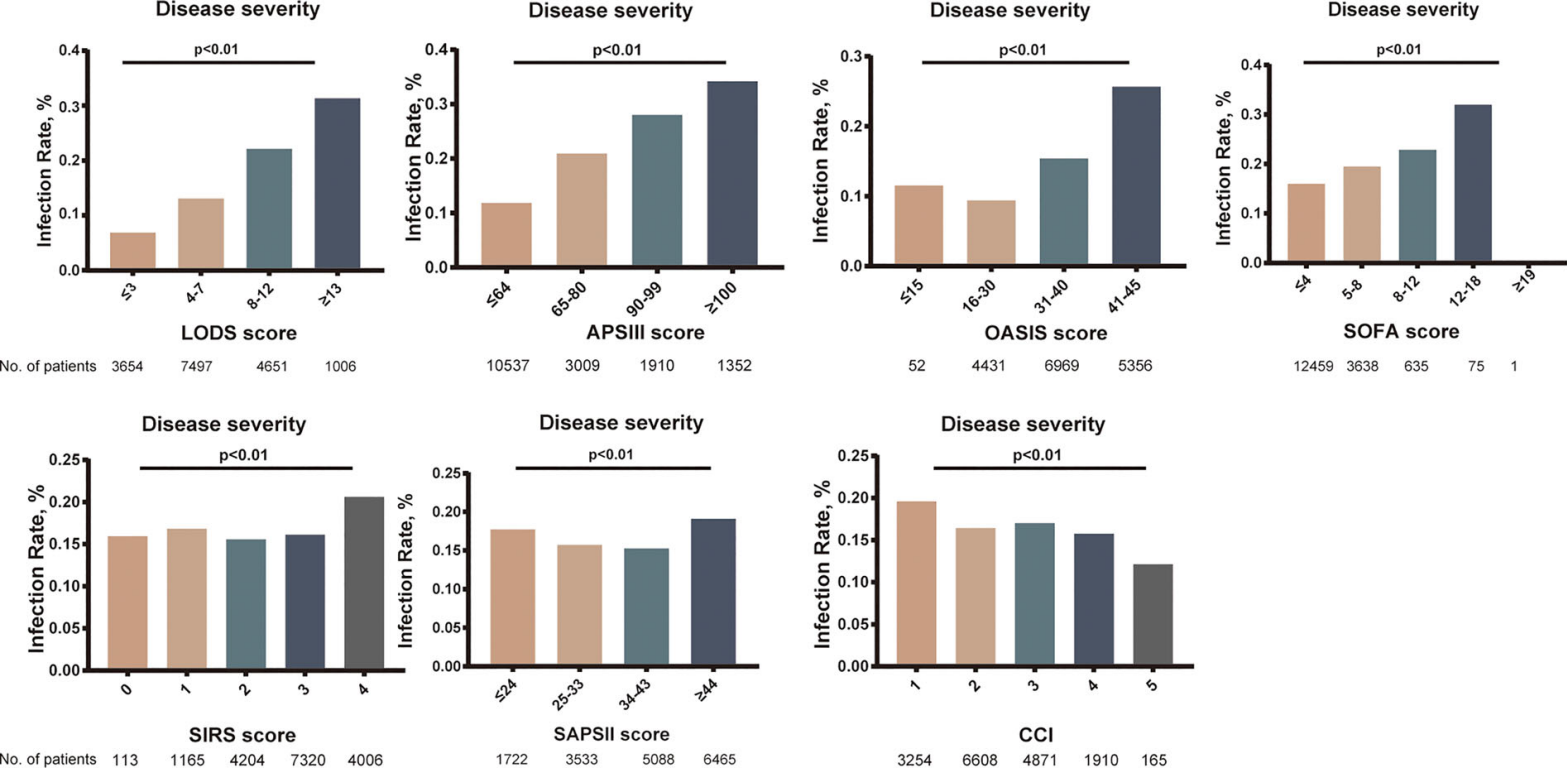
Relationship between the rate of intensive care unit (ICU)-acquired infection and disease severity. p < 0.001 for all panels.

In univariate analysis, in comparison with patients without ICU-acquired infection, patients who developed an ICU-acquired infection have higher SOFA score (4.05 ± 2.39 vs. 3.70 ± 2.07, p < 0.001), SIRS (2.90 ± 0.91 vs. 2.81 ± 0.89, p < 0.001), LODS (8.00 ± 3.51 vs. 6.07 ± 3.28, p < 0.001), SAPS II score (42.31 ± 15.04 vs. 40.63 ± 13.63, p < 0.001), APS III score (71.17 ± 27.28 vs. 56.83 ± 24.51, p < 0.001), and OASIS (40.02 ± 8.89 vs. 35.74 ± 8.88, p < 0.001). Subsequently, **Figure 3** found that the disease severity based on SIRS, SOFA, OASIS, LODS, APS III, CCI and SAPS II scores was greatly influenced by the incidence of ICU-acquired infections. We constructed models where the scoring system predicts an ICU-acquired infections. The AUROCs of the score system models’ predictive performance of the patients with an ICU-acquired infection in the training population are shown in **Table 3**.

**Table 3:** Comparison of score models between ICU-acquired infection and no ICU-acquired infection with sepsis.

Covariates	AUROC	95% confidence interval	p-value	Specificity	Sensitivity
LODS	0.655	0.642, 0.668	<0.001	0.603	0.635
APS III	0.657	0.644, 0.670	<0.001	0.612	0.624
OASIS	0.641	0.628, 0.654	<0.001	0.554	0.664
SIRS	0.529	0.515, 0.542	<0.001	0.773	0.28
SAPS II	0.535	0.521, 0.549	<0.001	0.57	0.499
SOFA	0.542	0.529, 0.556	<0.001	0.589	0.47
CCI	0.519	0.505, 0.533	<0.001	0.705	0.327

LODS, Logistic Organ Dysfunction Score; APS III, Acute Physiology Score III; OASIS, Oxford Acute Severity of Illness Score; SIRS, Systemic Inflammatory Response Syndrome Score; SAPS II, Simplified Acute Physiology Score II; SOFA, Sequential Organ Failure Assessment; CCI, Charlson Comorbidity Index.

Mutivariable logistic analysis in training cohort showed that mechanical ventalation (OR 2.64; 95% CI, 2.28–3.07), cerebrovascular insufficiency (OR 2.45; 95% CI, 2.01–2.98), tracheostomy (OR 2.11; 95% CI,1.14–3.90), anticoagulant medication (OR 1.95; 95% CI, 1.73–2.19), LODS (OR 1.57; 95% CI, 1.44–1.70), surgical ICU (OR 1.65; 95% CI, 1.49–1.83), Gram-negative bacteria (OR 1.29; 95% CI, 1.13–1.48), red blood cell (RBC) transfusion (OR 1.40; 95% CI, 1.26–1.57), central venous catheter (OR 1.12; 95% CI, 1.00–1.27), and urinary catheter (OR 1.31; 95% CI, 1.17–1.47) were independent predictors of developing ICU-acquired infection in septic patients (**Table 4**).

**Table 4:** Multivariate logistic analysis of risk factors for ICU-acquired infection in patients with sepsis.

Covariates	Training cohort		Validation cohort	
	Odds ratio (OR)	95% confidence interval (95% CI)	Odds ratio (OR)	95% confidence interval (95% CI)
Mechanical ventilation	2.64	2.28–3.07	2.69	2.15–3.36
Cerebrovascular insufficiency	2.45	2.01–2.98	1.77	1.30–2.41
Tracheostomy	2.10	1.13–3.90	5.84	2.48–13.77
Anticoagulant	1.95	1.73–2.19	2.08	1.73–2.51
SICU	1.65	1.49–1.83	1.89	1.61–2.22
LODS	1.57	1.44–1.70	1.78	1.57–2.02
RBC	1.40	1.26–1.57	1.31	1.11–1.55
Urinary catheter	1.31	1.17–1.47	1.28	1.06–1.53
Gram-negative bacteria	1.29	1.13–1.48	1.16	0.94–1.43
Central venous catheter	1.12	1.00–1.27	1.22	1.02–1.46

LODS, Logistic Organ Dysfunction System; SICU, surgical ICU; RBC, transfusion of red blood cells.

**Figure 4:**
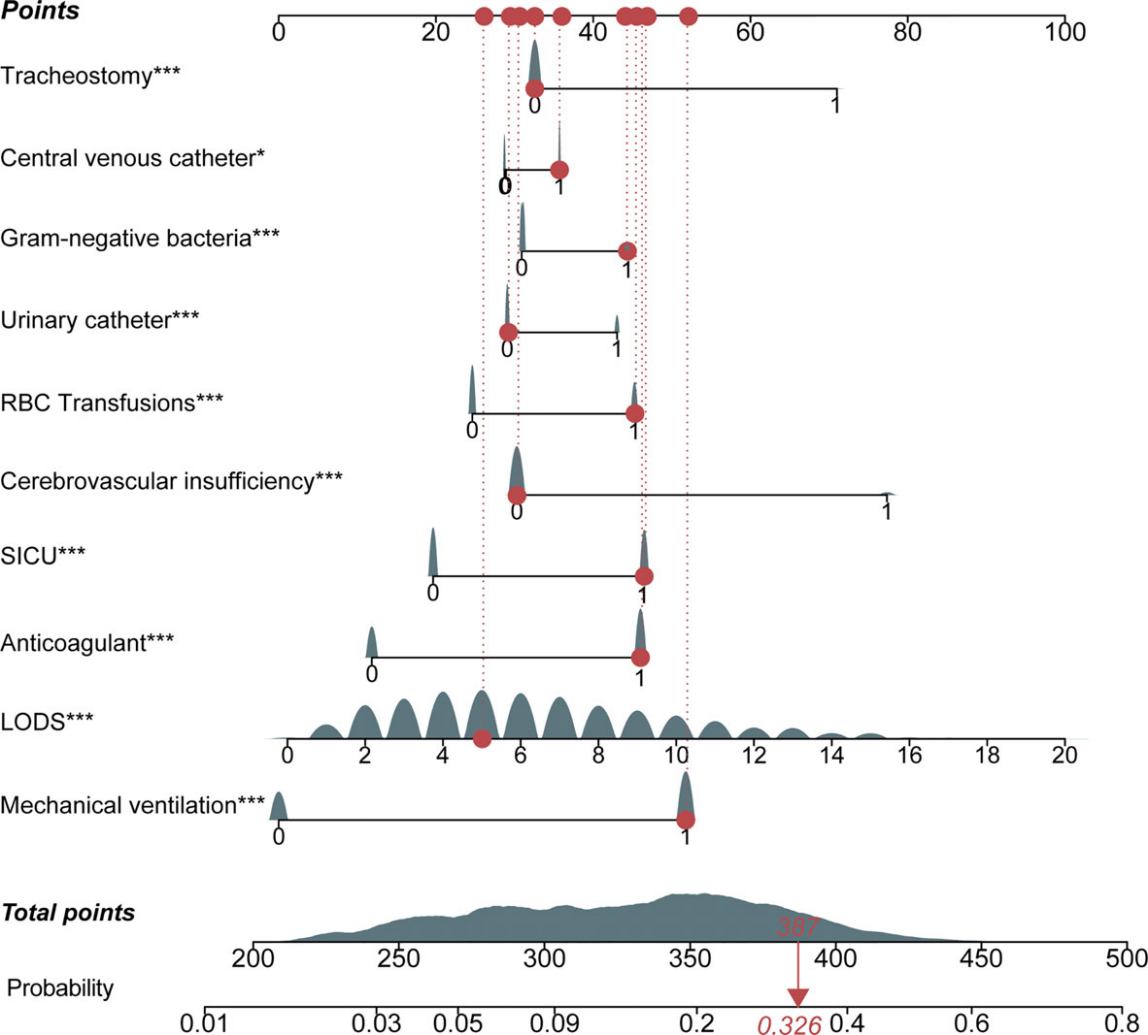
Nomogram for the prediction of intensive care unit (ICU)-acquired infections in sepsis. Each variable has corresponding points, and the total score for an individual patient could be obtained by summing up all scores. For categorical variables, 0 indicates “no,” while 1 means “yes.” The continuous variable, LODS, had continuous points. LODS, Logistic Organ Dysfunction Score; SICU, surgical ICU.

**Figure 5:**
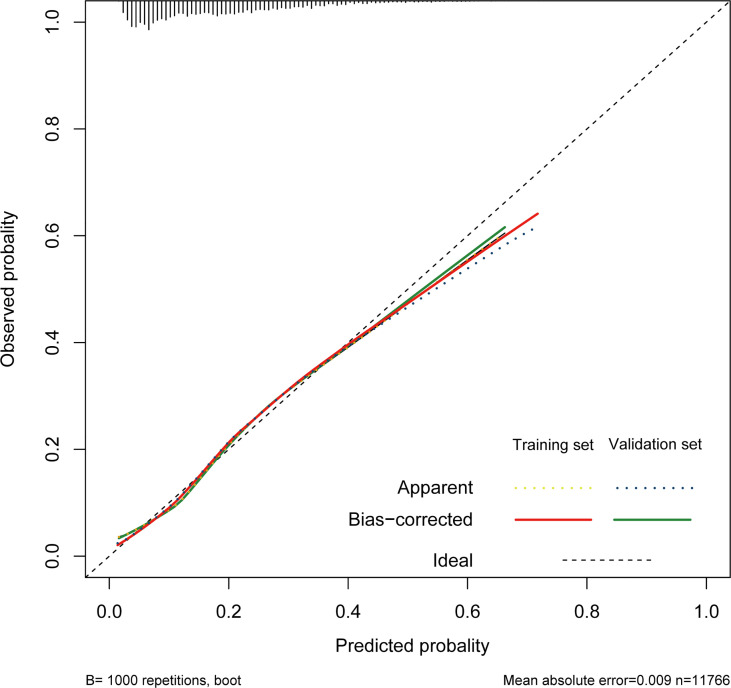
Calibration of the nomogram in the training cohort and validation cohort. The dotted line represents the ideal match between the nomogram-predicted survival (X-axis) and actual survival (Y-axis).

**Figure 6:**
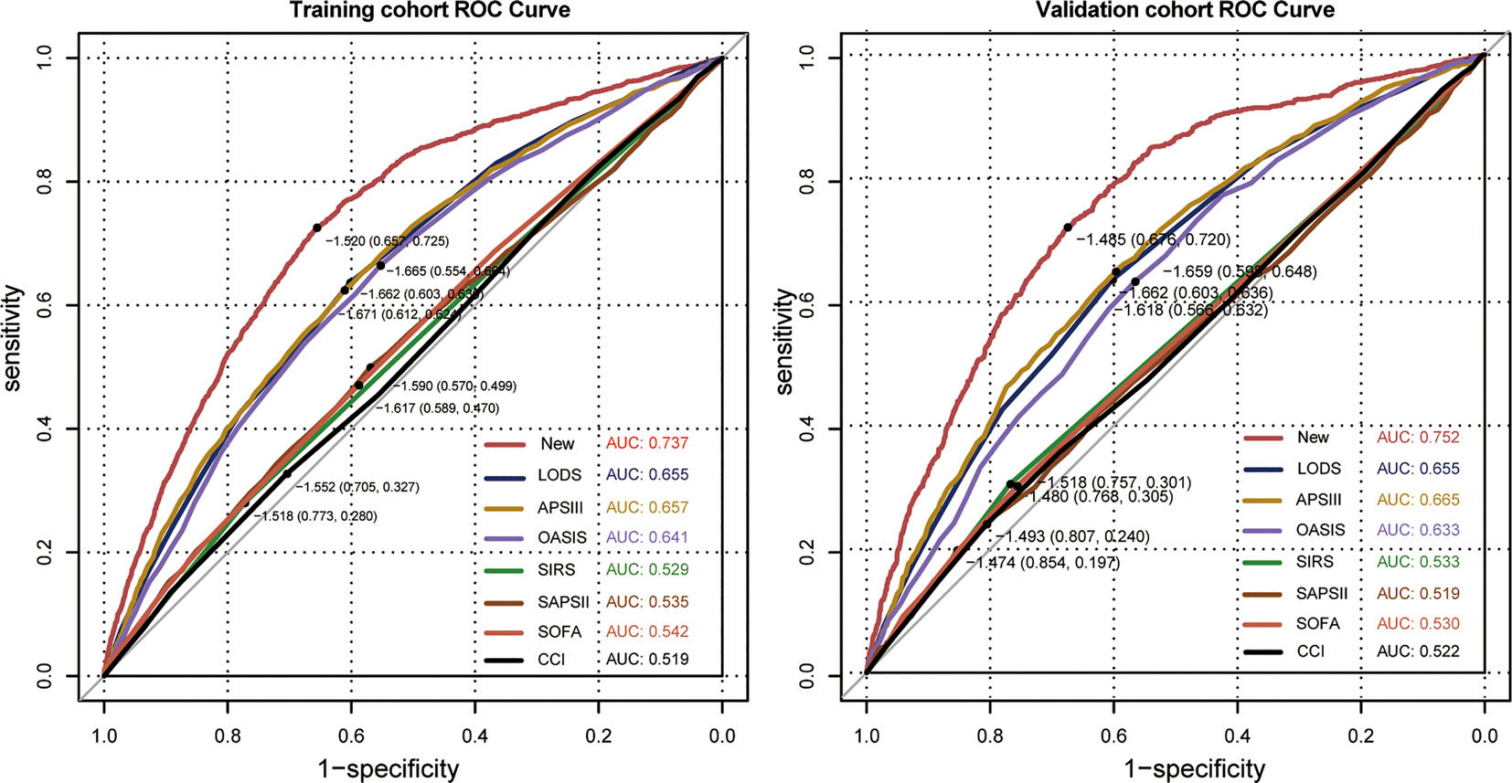
Corresponding receiver operating characteristic (ROC) curves of models in the training cohort and the validation cohort. The new model is the multivariate logistic regression model. LODS, Logistic Organ Dysfunction Score; APS III, Acute Physiology; OASIS, Oxford Acute Severity of Illness Score; SIRS, Systemic Inflammatory Response Syndrome Score; SAPS II, Simplified Acute Physiology Score II; SOFA, Sequential Organ Failure Assessment; CCI, Charlson Comorbidity Index.

**Figure 7:**
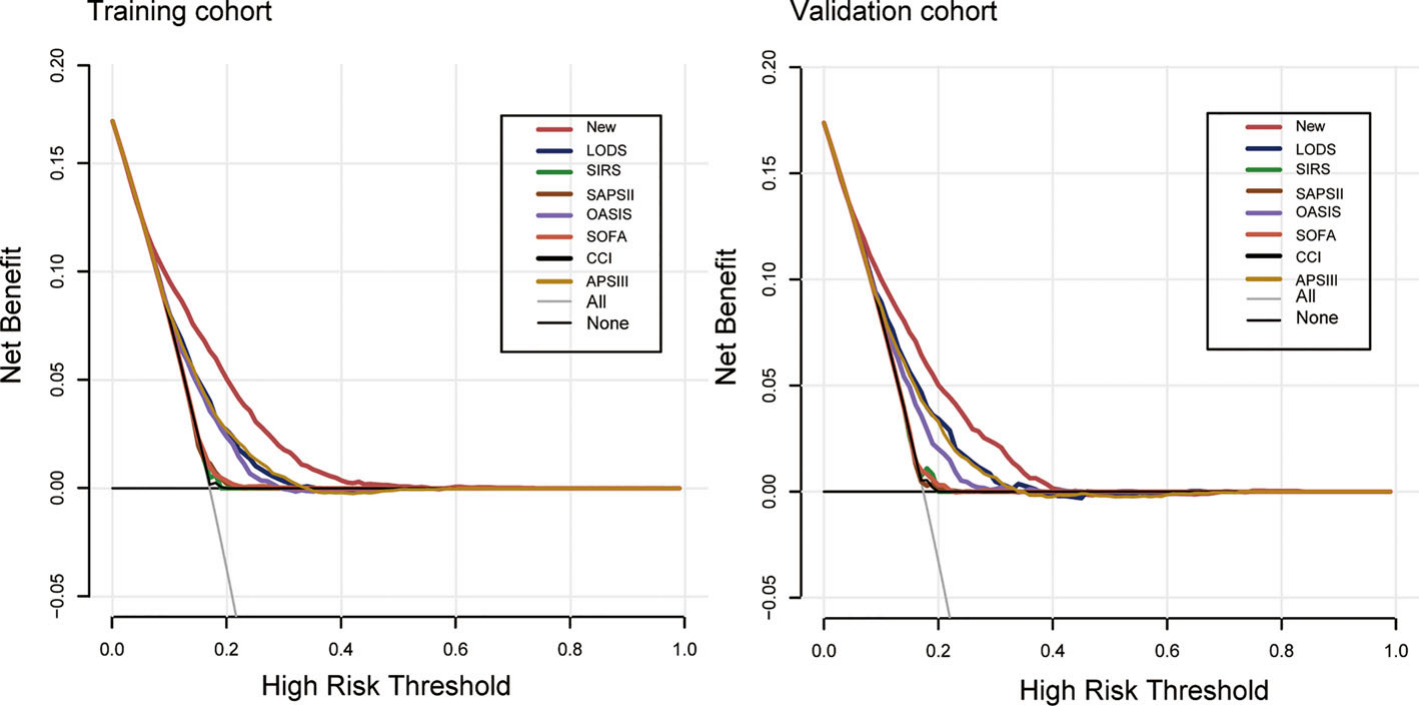
Decision curve analysis comparing the clinical utility of our new model (red line) with that of the other score systems. The clinical utility of all models is compared by measuring the net benefit (y-axis) for a range of threshold probability (x-axis). The black line represents the assumption that no patient has ICU-acquired infection. The gray line suggests that all patients have ICU-acquired infection. At the full range of displayed probabilities (0–0.2), the curve based on the integrated model (red) showed maximized benefit compared to other curves.

The nomogram of these predictors was presented in **Figure 4**. The C-index of the nomogram was 0.737 (95% CI, 0.725–0.749) in the training set and 0.752 (95% CI, 0.735–0.769) in the validation cohort. The calibration curves and the actual observations of probability were shown in **Figure 5**. The novel models had an AUC of 0.737 (95% CI, 0.725–0.749), 72.5% sensitivity, and 65.7% specificity (**Figure 6**). The novel models have a greater accuracy than that of the classical severity scoring systems alone in predicting ICU-acquired infection for septic patients (**Figure 6**). The decision curves of the clinical benefit of the new predictive models and all score system models were presented in **Figure 7**.

## Discussion

To the best of our knowledge, this is the first study using the large public database MIMIC IV to explore ICU-acquired infections in sepsis. Our study strictly followed the Sepsis-3 definition of suspected infection with organ dysfunction. Additionally, the database spanned a period of 10 years, far exceeding those of previous investigations, ensuring the practical significance in the context of an epidemic and showing feasibility in the real world.

We highlight that microbiological burden and mortality from ICU-acquired infection gradually increase over time in survivors of sepsis, despite higher mortality during the first 28 days in the non-ICU-acquired infection group. Severe infection triggers a cytokine storm, promoting widespread host responses, including microcirculation failure, organ dysfunction, shock, and death (Kaukonen et al., 2014). Early sepsis mortality has significantly decreased with the active implementation of the Surviving Sepsis Campaign (Coopersmith et al., 2018). Consequently, long-term outcomes after sepsis have attracted increasing attention. Two recent large studies had shown that ICU-acquired infection can greatly increase sepsis-related death, reporting rates of 44.2% and 49.2% by day 60 (van Vught et al., 2016; van Vught et al., 2017). While Goldenberg et al. (2014) reported that ICU-acquired infection only accounted for 14% of deaths in sepsis, with other potential contributors to sepsis mortality including mitochondrial dysfunction and microvascular leak. The incidence rate of ICU-acquired infections in sepsis was 17.1% in this study. Similar findings were reported in those two previous large observational studies, which were 14.4% and 13.5%, respectively, although we recorded a slightly higher cumulative incidence of ICU-acquired infections (van Vught et al., 2016; van Vught et al., 2017). Nevertheless, the higher observation was discovered in two retrospective studies, which were 31.0% and 38.9% (Zhao et al., 2016; Chen et al., 2019). This may have been due to the relatively limited sample size and the definition of sepsis 2.0.

For ICU patients, the SIRS, SOFA, OASIS, LODS, APS III, SAPS II, and CCI scores are fundamental individualized risk assessments for characterizing disease severity and degree of organ dysfunction (Minne et al., 2008; Chen et al., 2019; Li et al., 2020; Zhu et al., 2022). As is well known, severe sepsis may be related to immunosuppression. Immunosuppressed patients are highly susceptible to dangerous opportunistic pathogens and can rapidly succumb to infection (Sundar and Sires, 2013). Furthermore, the SAPS II, SIRS, and the degree of organ failure had been identified as poor early warning tools for the infection (Vincent et al., 2009; Herrod et al., 2018). Given the finding, we attempted to build regression models with these scoring systems and compare with our newly developed predictive model. The nomogram included the LODS providing the best discrimination capacity and a net benefit over any current common clinical scoring system models across all thresholds.

SOFA score was conceived as a tool to describe the occurrence of dysfunction in various organs and predict outcome, but several studies clarified that the prognostic values of the LODS or SAPS II were slightly better than those of SOFA scores in predicting sepsis mortality events (Li et al., 2020; Zhu et al., 2022). Consistent with this, the SOFA score in predicting the acquisition of infection, which increases the morbidity and mortality of patients with sepsis, shows a poorer prediction in the present study.

Being an adult accompanied by cerebrovascular insufficiency was an independent risk factor of ICU-acquired infection, especially traumatic brain injury, ischemic stroke, and intracerebral hemorrhage, which had not been reported in sepsis previously. The brain injury–induced immunosuppression syndrome initiated after acute brain injury and ischemia had been recognized from animal models, which was mediated by an intense activation of the hypothalamic-pituitary axis and the sympathetic nervous system (Dirnagl et al., 2007). Enhanced catecholamine levels and subsequent suppressed immune function are associated with the occurrence of infections after acute brain injury (Haeusler et al., 2008; Klehmet et al., 2009).

Almost 40% of patients undergoing craniotomy develop infection at least once, such as pneumonia independent of mechanical ventilation (15%), ventilator-associated pneumonia (VAP; 23%), or urinary tract infection (UTI; 9%), and postoperative surgical site infections (SSIs) occur in 9% (Kourbeti et al., 2015). Moreover, invasive procedures, including central venous catheter, urinary catheter, mechanical ventilation, and tracheostomy, are potential risk factors during the first 48 h in the ICU, predicting an ICU-acquired infection in our study due to the fact that these tubes provide an ideal opportunity for bacterial adhesion and biofilm formation (Sottile et al., 1986; Elsayed et al., 2022). The rapid colonization of microbiota contributes to the infection. Therefore, more research should focus on antimicrobial-coated catheter materials to prevent the occurrence of infection and progression.

Transfusion of RBCs had emerged as a potential risk factor for hospital-acquired infections in various clinical settings, including sepsis (Dupuis et al., 2017; Dupuis et al., 2017; Nederpelt et al., 2020). In addition, it is associated with an increased risk of venous thrombosis with allogeneic transfusion and transfusion-related infections (Jaffray et al., 2020; Miyamoto et al., 2021). Furthermore, stored RBCs and platelets can release intracellular cytokines and other components that induce an immunosuppressive phenotype in antigen-presenting cells and lymphocytes (Aslam et al., 2008; Torrance et al., 2015).

Notably, sepsis caused by Gram-negative bacillus infection enhances the risk of ICU-acquired infection. Among the 27,766 cases of central venous catheter-associated bloodstream infections reported to the National Healthcare Safety Network (NHSN) between 2009 and 2010, a large proportion of ICU-isolated drug-resistant bacteria are Gram-negative (Sader et al., 2014). Treatment resistance and non-susceptibility to antimicrobial agents lead to low treatment efficacy and complex therapeutic regimens. There is increasing awareness that elevated susceptibility to ICU-acquired infection can be pathogen-specific due to different patterns of immune barrier destruction, which can lead to opportunistic bacterial and fungal infections (Koch et al., 2017).

Although the use of a large dataset from a public database provides substantial support for our findings, our study has several limitations. First, our findings may have been influenced by confounding factors due to septic patients being admitted to the ICU with varying severities of illness, but the largest cohort study including patient data collected for almost 10 years should be clinically representative. Second, all of the variables included in our predictive model related mainly to patient demographic characteristics, underlying disease, pathogens, and supportive treatments, and our model lacked some meaningful laboratory metrics, leading to a low predictive efficiency of our model. However, all of the clinical information is available in lots of institutions, and a multivariable logistic risk model was used to evaluate the real clinical situations. Indeed, there were no significant differences in the levels of lymphocytes, neutrophils, partial thromboplastin time (PT), or activated partial thromboplastin time (APTT) in our cohort. Microcirculation disorders occur early, 12 h after the sepsis, and are associated with neutrophil–endothelium interactions, microthrombus formation, and vascular leakage (Wu et al., 2015). This may explain that the indicators of coagulation function did not differ between those with ICU-acquired infection and those without. Additionally, the occurrence of acquired infection is mainly related to immune cell function; the value of some biomarkers of immune cell function in predicting susceptibility to ICU-acquired infections warrants exploration (van Vught et al., 2016). Third, up to dozens of risk factors were included in our model, which may be related to the heterogeneity of the group with sepsis. To overcome this limitation, we strictly followed the latest diagnostic criteria for sepsis and comprehensively accounted for pathogens, antibiotics, and other relevant factors, which may have improved the prediction of secondary infection occurrence.

## Conclusion

Septic survivors are burdened with an elevated risk of ICU-acquired infections, and the acquired infections resulted in an increase in long-term mortality. Patients with cerebrovascular insufficiency or Gram-negative bacteria, in the surgical ICU, undergoing management of the central venous catheter or urinary catheter, mechanical ventilation, tracheostomy, or RBC transfusion, and receiving anticoagulant medication within 48 h after ICU admission were much more likely to develop ICU-acquired infection. The findings may assist physicians in optimizing the management of sepsis-related ICU-acquired infection and improve the prognosis of sepsis.

## Data availability statement

The original contributions presented in the study are included in the article/supplementary material. Further inquiries can be directed to the corresponding author.

## Ethics statement

The MIMIC-IV database was approved by the Massachusetts Institute of Technology (Cambridge, MA) and Beth Israel Deaconess Medical Center (Boston, MA).

## Author contributions

YH extracted the data from the MIMIC-IV database. JX, YH, and YS contributed to literature search, and drafted the manuscript. YS and JX contributed to this work and designed the study. All authors contributed to the article and approved the submitted version.

## Funding

This study was supported by National Natural Science Foundation of China (No. 82002026, 81971818, and 81772047), and the National Key Research and Development Project (2021YFC2500802). The funders had important role in the design of the study, in the collection, analysis, and interpretation of the data, or in the writing of the manuscript.

## Acknowledgments

We would like to thank the Massachusetts Institute of Technology and the Beth Israel Deaconess Medical Center for the MIMIC project.

## Conflict of interest

The authors declare that the research was conducted in the absence of any commercial or financial relationships that could be construed as a potential conflict of interest.

## Publisher’s note

All claims expressed in this article are solely those of the authors and do not necessarily represent those of their affiliated organizations, or those of the publisher, the editors and the reviewers. Any product that may be evaluated in this article, or claim that may be made by its manufacturer, is not guaranteed or endorsed by the publisher.

## Abbreviations

ICU, intensive care unit; MIMIC, Medical Information Mart for Intensive Care; LOS, length of stay; IQR, interquartile range; AUROC curve, area under the receiver operating characteristic curve; OR, odds ratio; CI, confidence interval; MRSA, methicillin-resistant *Staphylococcus aureus*; RBC, red blood cell; SD, standard deviation; SOFA, Sequential Organ Failure Assessment; SIRS, Systemic Inflammatory Response Syndrome Score; OASIS, Oxford Acute Severity of Illness Score; LODS, Logistic Organ Dysfunction Score; SAPS II, Simplified Acute Physiology Score II; APS III, Acute Physiology Score III; CCI, Charlson Comorbidity Index.
